# Supplementary figures and images for: Heterologous booster with a novel formulation containing glycosylated trimeric S protein is effective against Omicron
Source: Front Immunol. 2023 Nov 10;14:1271209. doi: 10.3389/fimmu.2023.1271209 (PMC10667599; doi:10.3389/fimmu.2023.1271209)

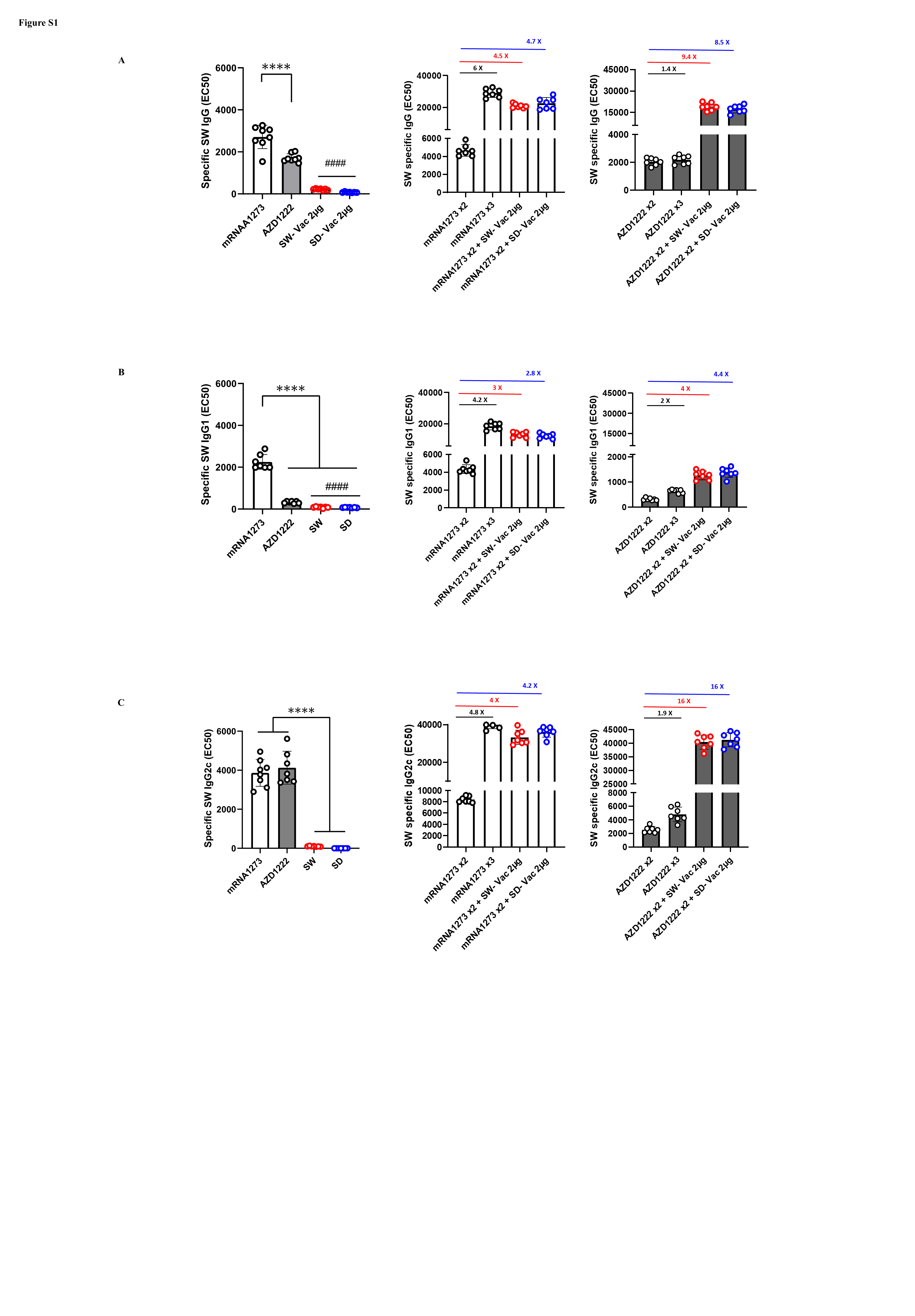

Supplement: Supplementary Figure 1 — Specific ancestral Spike (SW) IgG and IgG isotypes levels induced by homologous and heterologous vaccination schedule. SW-specific IgG (A), IgG1 (B), and IgG2c (C) levels induced by either a single dose of commercial vaccines mRNA-1273, AZD1222/ChAdOx1, SW-Vac 2μg, or SD-Vac 2μg (right panel) or by a 2-dose primary series with or without homologous or heterologous boosters (middle and left panels). Serum collected on Day 14 after the last dose was analyzed by ELISA. Serum antibody levels are expressed as EC50 ± SD for each group. At the top of the figures, the increases between the levels found for homologous or heterologous boosters versus those detected after the primary regimen are indicated. For statistical analysis, antibody levels were assessed using a one-way ANOVA with Bonferroni’s multiple comparisons test. The * symbol indicates significant differences among immunized groups: ****p<0.0001, ***p<0.001. The # symbol indicates significant differences between antibodies levels detected for SW-Vac 2μg or SD-Vac 2μg-immunized animals and those from animals immunized with commercial vaccines: #### p<0.0001. [file Image_2.tif]

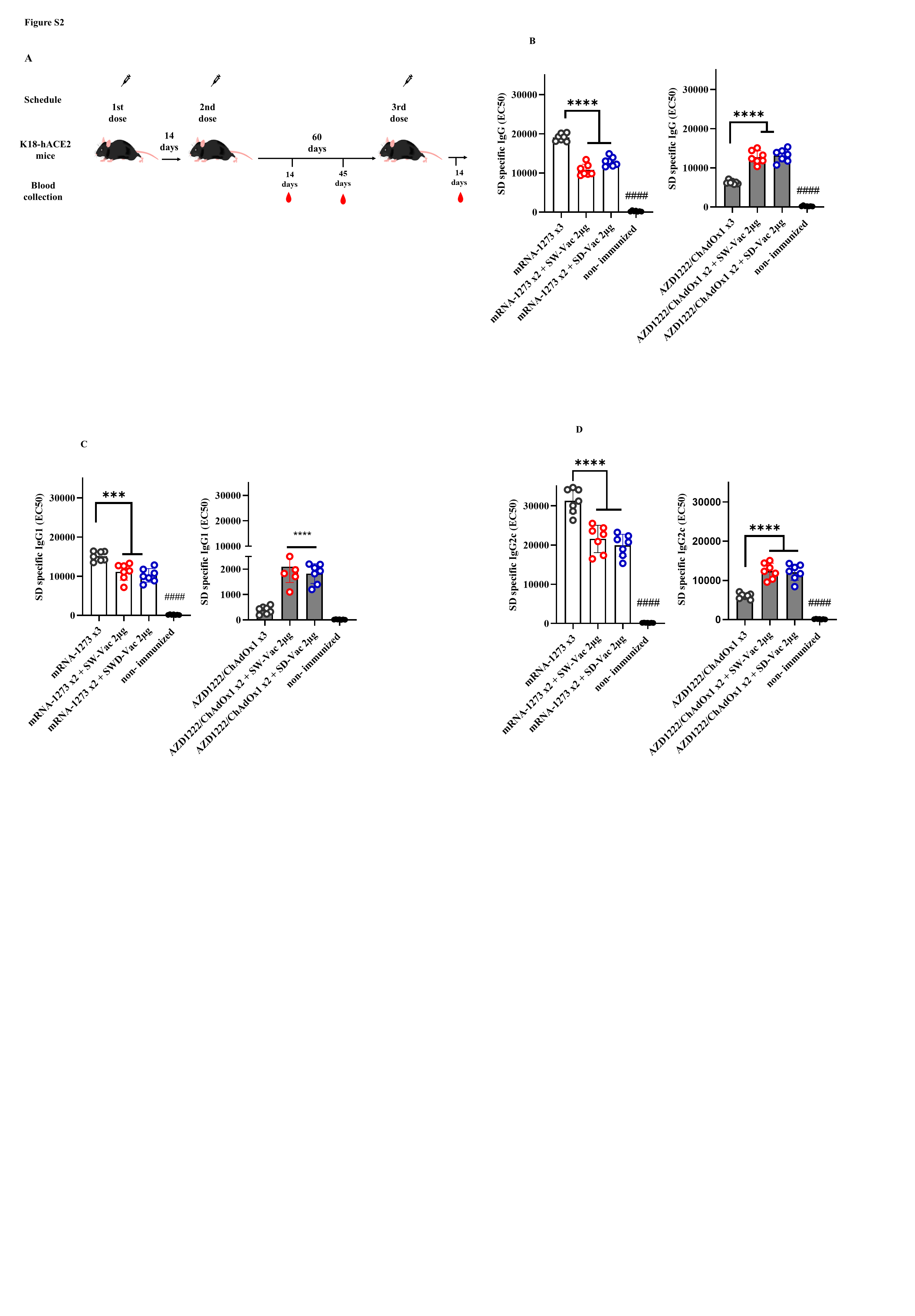

Supplement: Supplementary Figure 2 — Specific Spike (SD) IgG and IgG isotypes levels induced by homologous and heterologous vaccination schedule. (A) Mice vaccination schedule K18-hACE2 mice (n =7/group) were immunized on days 0 and 14 with anti SARS-CoV-2 commercial vaccines mRNA-1273 or AZD1222/ChAdOx1 and boostered at day 60 with formulations containing purified spike protein from ancestral (SW) or Delta (SD) SARS-CoV-2 variant delivered intramuscularly. SD specific (B) IgG, (C) IgG1 and (D) IgG2c levels induced by 3-dose homologous and heterologous schemes. Sera from immunized and non-immunized were collected 14 days after the 3rd dose and analyzed by ELISA. Serum antibody levels are expressed as EC50 ± SD for each group. For statistical analysis, antibody levels analyzed by a one-way ANOVA with Bonferroni’s multiple comparisons test. The * symbol indicate significant differences among immunized: ****p<0.0001, *** p<0.001, *p<0.05. The # symbol indicate significant differences between non-immunized and immunized animals: #### p <0.0001. [file Image_3.tif]

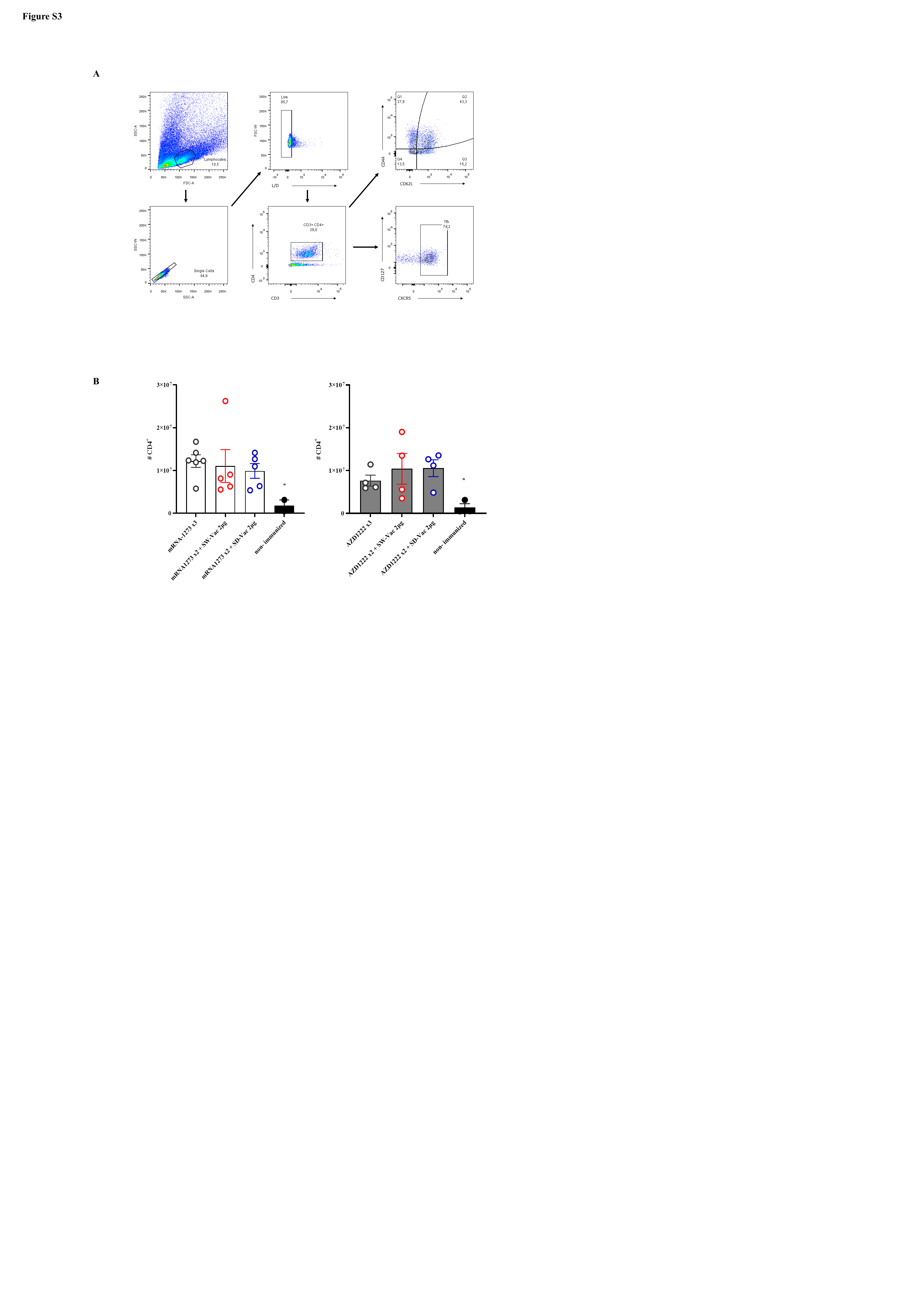

Supplement: Supplementary Figure 3 — CD4+ populations induced after Omicron BA.1 challenge in mice vaccinated with homologous or heterologous schemes. Five days post challenge immunized and non-immunized mice were sacrificed and spleen cells were collected. Cells were incubated with LIVE/DEAD Violet (Invitrogen), followed by surface staining with fluorochrome-conjugated anti-mouse Abs for CD4+ T cells. (A) Gate strategy for CD4+ population analysis by flow citometry. CD4+ effector T cells (CD3+CD4+CD44+CD62L-). CD4+ central memory T cells (CD3+CD4+CD44+CD62L+). CD4+ follicular T cells (CD3+CD4+CXCR5+CD127Low/high). Flow cytometry analysis was performed on an BD FACS Aria Fusion. The results were analyzed using Flow Josoftware (TreeStar). (B) Total number of CD4+ cells induced after Omicron BA.1 challenge in mice vaccinated with homologous or heterologous schemes. The represented data correspond to the mean of absolut cells counts ± SEM. Two-way ANOVA followed by Bonferroni post-test were used for statistical analysis. *p < 0.05. [file Image_4.tif]

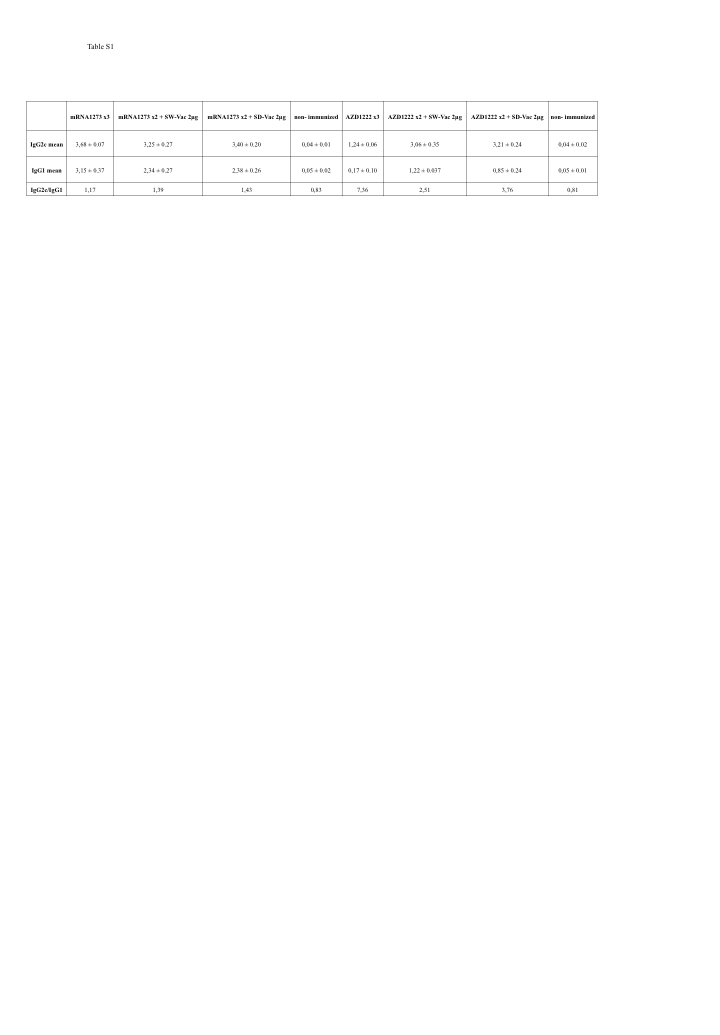

Supplement: Supplementary Table 1 — IgG2c and IgG1 titters and IgG2c/IgG1 ratio for homologous and heterologous immunized mice. Data for non-immunized mice are also included. [file Image_1.tiff]
